# Supplementary material for: In Vitro Investigations on Optimizing and Nebulization of IVT-mRNA Formulations for Potential Pulmonary-Based Alpha-1-Antitrypsin Deficiency Treatment
Source: Pharmaceutics. 2021 Aug 17;13(8):1281. doi: 10.3390/pharmaceutics13081281 (PMC8399093; doi:10.3390/pharmaceutics13081281)
Supplement: Supplementary file 1 [file pharmaceutics-13-01281-s001.zip › pharmaceutics-1327253-supplementary.pdf]

pharmaceutics

MDPI

Supplementary Materials: In Vitro Investigations on Optimizing and Nebulization of IVT-mRNA Formulations for Potential Pulmonary-Based Alpha-1-Antitrypsin Deficiency Treatment

Shan Guan, Max Darmstädter, Chuanfei Xu and Joseph Rosenecker

This File Includes:

Supplementary Table S1
Supplementary Figure S1 to S3

Supplementary Table

Table S1. Size of IVT-mRNA/Lipofectamine2000 lipoplexes using Protocol 2.

Amounts of Lipofectamine2000 (µL)	Amounts of IVT-mRNA (µL)	Size (nm) Non-nebulized	Size (nm) Nebulized
7.2	18.0	564±73	798±102
10.8	18.0	748±165	820±195
14.4	18.0	804±203	840±130

The complexes were prepared in OptiMEM. After a 10 minute incubation period the size measurement was performed. The data represent hydrodynamic diameter±SD, n=3.

Supplementary Figures

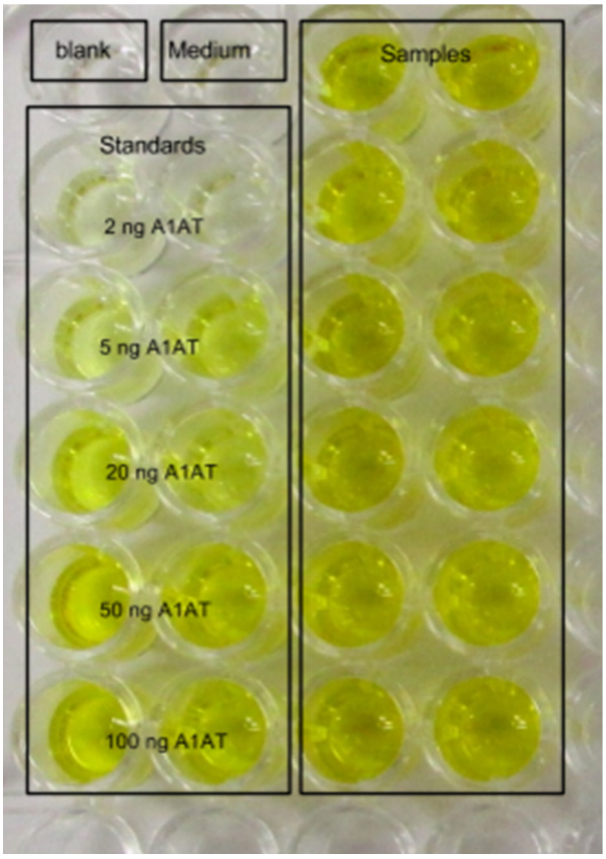

Figure 1S. A representative ELISA microplate of A1AT-mRNA/Lipofectamine2000 complexes mediated transfection. 24 h after transfection, 16HBE cell culture supernatants were collected and

centrifuged at 3000 × g for 10 minutes to remove debris. 50 µl of each sample was pipetted into an ELISA plate. 2 ng, 5 ng, 20 ng, 50 ng and 100 ng of A1AT-standards were used.

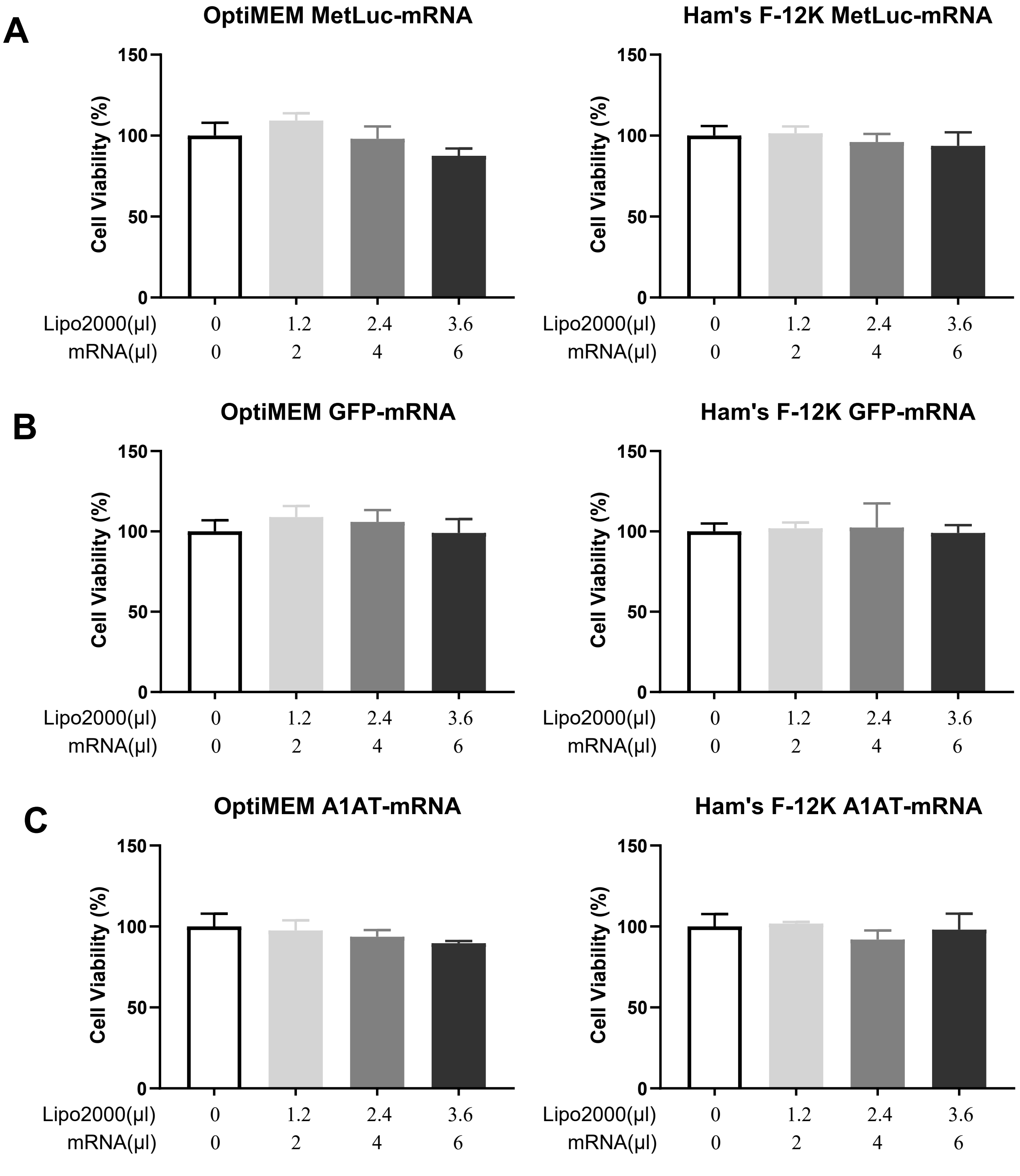

Figure S2. Cytotoxicity profile of IVT-mRNA/Lipofectamine2000 complexes towards 16HBE cells. The complexes were prepared in OptiMEM or Ham's F-12K medium by mixing indicated amounts of Lipofectamine2000 with MetLuc-mRNA (**A**) or GFP-mRNA (**B**) or A1AT-mRNA (**C**) at the concentration of 0.1 µg/µl, respectively. The complexes were incubated with 16HBE cells for 2 h. Cell viability was assayed 24 h after transfection via an MTT assay, untreated cells were used as a control (100%), n=6. "Lipo2000" represents Lipofectamine2000 and "mRNA" represents MetLuc-mRNA (**A**) or GFP-mRNA (**B**) or A1AT-mRNA (**C**).

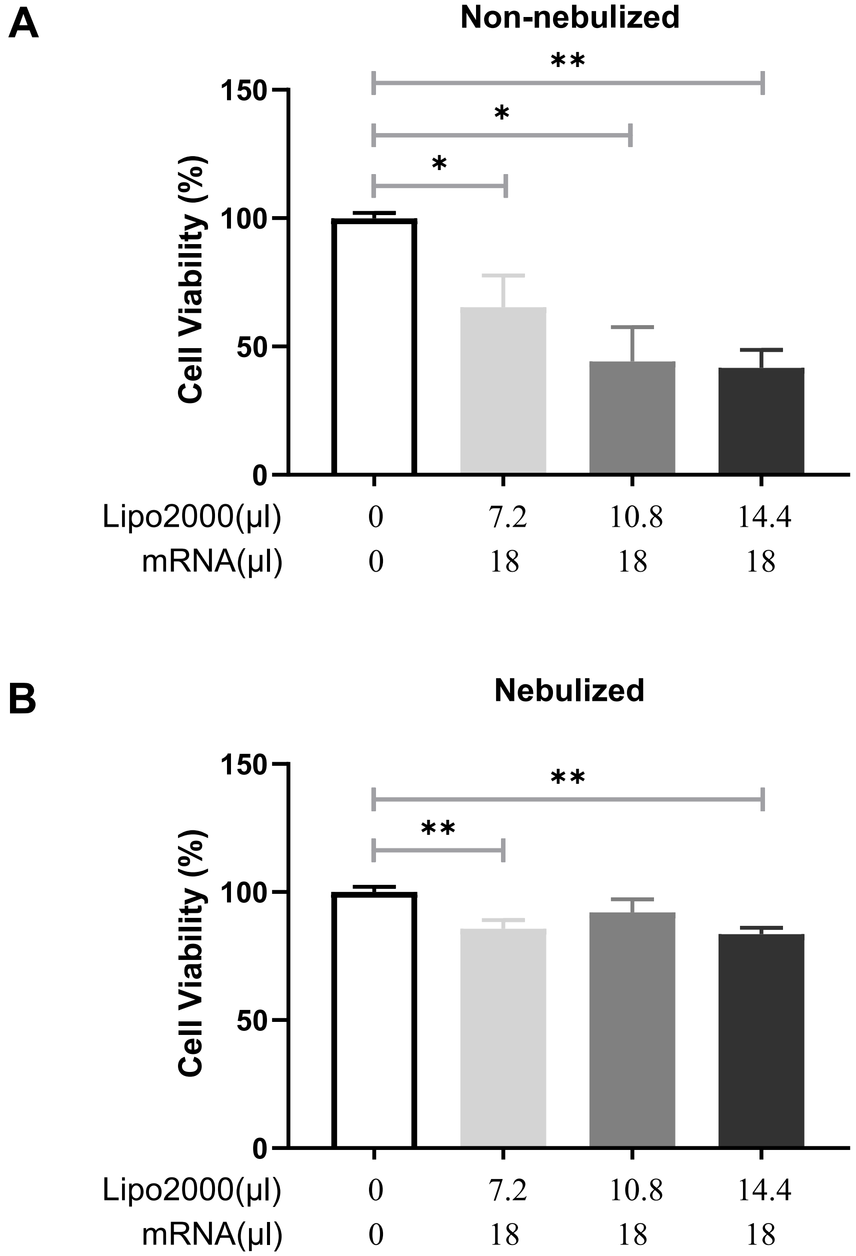

Figure 3. Cytotoxicity profile of MetLuc-mRNA/Lipofectamine2000 complexes prepared by Protocol 2 using Ham's F-12K medium towards 16HBE cells. 7.2 µl, 10.8 µl and 14.4 µl Lipofectamine2000 was mixed with 18 µl MetLuc-mRNA. "Lipo2000" represents Lipofectamine2000 and "mRNA" represents MetLuc-mRNA. A fraction of the complexes was kept separately and used as a "non-nebulized" sample (**A**). The rest of the solution was aerosolized for 5 minutes using a PARI Boy® jet-nebulizer and the collected part was used as a "nebulized" formulation (**B**). The complexes were incubated with 16HBE cells for 2 h. Cell viability was assayed 24 h after transfection. Untreated cells were used as 100%, n=6. The results were analysed for the statistical significance with a two-tailed unpaired student's t-test: * $p< 0.05$ and ** $p < 0.01$.
